# Prescribing trends of cardioprotective medications among patients with diabetes and ASCVD in primary care setting in Qatar

**DOI:** 10.1080/20523211.2025.2594824

**Published:** 2025-12-08

**Authors:** Myriam Jaam, Ahmed Awaisu, Daoud Al-Badriyeh, Amani Zidan, Rana Abu Samaha, Basant Elkattan, Zanfina Ademi, Mohammed Ghaith Al-Kuwari, Palli Valappila Abdul Rouf, Jazeel Abdulmajeed, Dina Abushanab

**Affiliations:** a Department of Clinical Pharmacy and Practice, College of Pharmacy, QU Health, Qatar University, Doha, Qatar; b Health Economics and Policy Evaluation Research (HEPER) Group Centre for Medicine Use and Safety, Faculty of Pharmacy and Pharmaceutical Sciences, Monash University, Melbourne, Australia; c Strategy Planning & Health Intelligence, Primary Healthcare Corporation, Doha, Qatar; d College of Medicine, QU Health, Qatar University, Doha, Qatar; e Clinical Trial Unit, Hamad Medical Corporation, Doha, Qatar; f Department of Pharmacy, Hamad Medical Corporation, Doha, Qatar

**Keywords:** Prescribing pattern, diabetes, ASCVD, high-intensity statins, SGLT2i, GLP-1RA, ACEi, ARBs

## Abstract

**Background:**

Despite established guidelines recommending cardioprotective medications for patients with type 2 diabetes mellitus (T2DM) and atherosclerotic cardiovascular disease (ASCVD), prescribing practices often fall short of recommendations. In this study we aimed to assess the prescribing trends of guidelines-recommended cardioprotective medications, including sodium-glucose cotransporter-2 inhibitors (SGLT2i), glucagon-like peptide-1 receptor agonists (GLP-1RA), angiotensin-converting enzyme inhibitors/angiotensin receptor blockers (ACEi/ARB), and high-intensity statins, among patients with type 2 diabetes mellitus (T2DM) and atherosclerotic cardiovascular disease (ASCVD) in Qatar's primary health care corporation (PHCC).

**Methods:**

This retrospective cross-sectional study analysed electronic medical records data from 29 PHCC centres in Qatar for the period January-December 2021. Prescription rates of SGLT2i/GLP-1RA, ACEi/ARB, and high-intensity statin were assessed based on documented medication usage. Logistic regression was used to identify factors associated with guideline-directed prescribing. The study received ethics approval from the PHCC in Qatar, with de-identified data accessible only to the Principal Investigator.

**Results:**

Among the 2,870 eligible subjects, only 10% (*n* = 292) were prescribed high-intensity statins, 12% (*n* = 357) received SGLT2i/GLP-1RA, and 56% (1,617)were prescribed ACEi/ARB. Male gender, higher HbA1c, and a diagnosis of dyslipidaemia were positively associated with increased likelihood of adherence to relevant clinical practice guidelines. However, these factors only explained only 16% of the observed variance.

**Conclusion:**

The majority of patients with T2DM and ASCVD did not receive the completed guideline-recommended regimens of cardioprotective medications, suggesting a significant gap in clinical practice. Urgent implementation of multifaceted strategies is warranted to address barriers and clinical inertia, thereby optimising cardiovascular risk reduction and secondary prevention therapies.

## Background

Diabetes remains a major public health concern globally. According to the International Diabetes Federation (IDF), over 589 million adult people were diagnosed with diabetes in 2024, with the Middle East and North African (MENA) region accounting for 84.7 million cases, a figure projected to rise by 92% by 2050 (International Diabetes Federation, 2025). The IDF has highlighted diabetes as one of the most rapidly growing global health crises of the twenty-first century. Qatar, a country in the MENA region with a population of approximately 2.8 million people, currently has a diabetes prevalence of 18.7%, which translates to around 409,300 confirmed cases (International Diabetes Federation, 2025; World Health Rankings, 2020). This prevalence significantly exceeds the global average of 11.1% and ranks among the highest in the MENA region, where countries like Saudi Arabia (21.4%), Kuwait (28.4%), and UAE (16.5%) also face similar challenges. This elevated prevalence across the Gulf region is associated with the sedentary lifestyles, dietary shifts toward processed foods, high obesity rates, and genetic predisposition within these populations (Awad et al., 2022; International Diabetes Federation, 2025). According to a recent epidemiological health assessment by the Primary Health Care Corporation (PHCC) in Qatar in 2019, the prevalence of type 2 diabetes among registered patients ranged from 12% to 14% across the three operational regions (western, northern, and central). This finding suggests that a substantial proportion of patients with diabetes receive care through the noncommunicable disease clinics of the primary healthcare centres in the country (Al-Kuwari et al., 2021). Qatar faces projected increases in diabetes-related health expenditure, which is expected to rise by 200–600% and potentially expected to account for up to 32% of total health expenditure by 2050 (Awad et al., 2018). Diabetes contributes substantially to several complications, with about 50% of end-stage renal disease (ESRD) cases requiring dialysis, about 50% of acute coronary syndrome (ACS) cases, and about 70% of stroke and transient ischemic attack cases, making it the second leading cause of death in Qatar after coronary heart disease (CHD) (International Diabetes Federation, 2025; Ministry of Public Health, 2022; World Health Rankings, 2020). Studies indicate that individuals with diabetes have twice the risk of CHD, 2.3 times the risk of ischemic stroke, and 1.7 times the risk of other vascular deaths compared to those without diabetes (Sarwar et al., 2010).

To mitigate the risks of morbidity and mortality associated with diabetes, the American Diabetes Association (ADA) strongly recommends using sodium – glucose cotransporter 2 inhibitor (SGLT2i) or glucagon-like peptide 1 receptor agonist (GLP-1RA) as part of the glucose-lowering treatment regimen for individuals with type 2 diabetes who also have established atherosclerotic cardiovascular disease (ASCVD). This recommendation is supported by multiple landmark randomised controlled trials (RCTs) demonstrating significant reductions in major adverse cardiovascular events (MACE), including heart failure hospitalisations and cardiovascular death, among ASCVD patients (Association, 2021, 2022; ElSayed et al., 2023; “Standards of Medical Care in Diabetes-2020 Abridged for Primary Care Providers”, 2020). Specifically, RCTs such as the EMPA-REG (Zinman et al., 2015), CANVAS (Neal et al., 2017), DECLARE-TIMI 58 (Wiviott et al., 2019), and HARMONY Outcomes (Hernandez et al., 2018) have shown cardiovascular benefits with SGLT2i, while LEADER (Marso et al., 2016), SUSTAIN-6 (Marso et al., 2016), and REWIND (Gerstein et al., 2019) trials have demonstrated similar benefits with GLP-1RA.

Patient-specific factors – such as renal function, cost and insurance coverage, risk of side effects, and individual preferences – should inform the choice between SGLT2i and GLP-1RA therapies (Association, 2021, 2022; ElSayed et al., 2023; “Standards of Medical Care in Diabetes-2020 Abridged for Primary Care Providers”, 2020). Additionally, ADA guidelines recommend prescribing angiotensin-converting-enzyme inhibitors (ACEi) or angiotensin 2 receptor blockers (ARBs) and high-intensity statins (defined as atorvastatin 40–80 mg daily or rosuvastatin 20–40 mg daily) for ASCVD patients to confer additional cardiovascular protection (Association, 2021, 2022; ElSayed et al., 2023; “Standards of Medical Care in Diabetes-2020 Abridged for Primary Care Providers”, 2020). Since 2020, the ADA guidelines have extended these recommendations (SGLT2i/GLP-1RA, ACEi/ARB) to include all people with ASCVD, regardless of their glycated hemoglobin (A1C) level or achievement of glycemic targets. This is because the cardiovascular benefits have been shown to be independent of glucose lowering benefits (Association, 2021, 2022; ElSayed et al., 2023; “Standards of Medical Care in Diabetes-2020 Abridged for Primary Care Providers”, 2020).

Despite the strong recommendations and the proven cardiovascular benefits of these medications, their utilisation remains suboptimal in real-world settings, with studies reporting that only 4.1% to 19% of eligible patients receiving recommended cardiovascular protective treatments (Korayem et al., 2022; Mody et al., 2022; Pantalone et al., 2018). This underutilisation is particularly concerning given the overwhelming evidence supporting the use of the medications in reducing cardiovascular risk among people with diabetes and established ASCVD. For instance, in a study involving over 500,000 patients with diabetes and ASCVD, only 11.2% received an SGLT2i and 8% received GLP-1RA, highlighting the urgent need for optimising treatment regimens (Mahtta et al., 2022). Similarly, the under-prescribing of high-intensity statins among ASCVD patients is also prevalent, ranging from 27–50%, despite their well-established benefits in reducing low-density lipoprotein (LDL) cholesterol levels and preventing cardiovascular events (Ahmed et al., 2021; Bideberi & Mutagaywa, 2022; Nelson et al., 2022)

This study aimed to determine whether the prescribing practices of cardioprotective agents for individuals with diabetes and established ASCVD in Qatar align with or surpass global trends. The objective was to evaluate adherence to evidence-based medicine (EBM) guidelines for prescribing these cardioprotective medications (including high-intensity statins, ACEi or ARB, and SGLT2i or GLP-1RA) in Qatar. Understanding current prescribing patterns is crucial to identifying gaps between guidelines and real-world clinical practice, and to assess whether Qatar's performance is on par with or exceeds international standards. This knowledge can inform future quality improvement and educational efforts to optimise the use of available beneficial therapies and ultimately improve individual outcomes in Qatar and beyond. By optimising the use of these effective therapies, this study ultimately aims to improve health outcomes for individuals in Qatar and potentially set a benchmark for other regions as well.

## Methods

### Context

Qatar's primary healthcare system is organised through the Primary Health Care Corporation (PHCC), which operates 29 health centres across three operational regions (western, northern, and central). Patients are typically registered with a specific PHCC centre based on their residential area, though they can access services at other centres when needed (e.g. in cases of simple emergencies). The PHCC functions as the first point of contact for healthcare services, with general practitioners being able to refer to specialists and to hospital care. The PHCC offers a wide range of services including noncommunicable disease clinics where patients diagnosed with chronic diseases such as diabetes are managed.

Under the Ministry of Public Health coverage system, all medications are fully covered for Qatari nationals in public sector facilities. However, at the time of this study, non-Qatari residents received up to 80% coverage with a 20% co-payment requirement for medications, including high-cost therapies, which was typically paid out-of-pocket (Nadeem et al., 2021).

For medication prescribing, PHCC physicians can prescribe both standard medications and newer therapies like SGLT2i/GLP-1RA This organisational structure means our study captures prescribing patterns from the primary care setting where most diabetes management occurs, making our findings representative of front-line clinical practice in Qatar's healthcare system.

### Study design and data source

This retrospective observational study utilised data extracted from electronic medical records across all Primary Health Care Corporation (PHCC) centres in Qatar (*N* = 29). The data collection period was from 1 January, 2021 to 31 December, 2021. Data were extracted from electronic medical records of people diagnosed with type 2 diabetes mellitus and established ASCVD. ASCVD was defined as having obstructive coronary artery disease, prior myocardial infarction, prior coronary revascularization (such as percutaneous coronary intervention or coronary artery bypass grafting), peripheral arterial disease, or cerebrovascular disease. Patients with type 2 diabetes mellitus were identified either through physician documentation in the electronic medical record or via HbA1c values that align with a diabetes diagnosis. Formal diagnostic coding systems (such as ICPC-2 or ICD-10) were not consistently present in the dataset, and case identification did not depend solely on prescribed medications. Also, ASCVD was identified through physician documentation in the electronic medical record, specifying a qualifying diagnosis such as obstructive coronary artery disease, prior myocardial infarction, prior coronary revascularization (e.g. PCI or CABG), peripheral arterial disease, or cerebrovascular disease. Formal diagnostic codes (ICPC-2, ICD-10, SNOMED-CT) were not available in the dataset.

### Data collection

Data collection for this study involved retrieving information from electronic medical records of eligible individuals at all PHCC centres in Qatar. Our data collection was based on medications documented as prescribed in electronic medical records rather than pharmacy dispensing data or patient-reported medication use. This includes medications that were electronically prescribed by PHCC physicians and recorded in the patient's current medication list within the electronic health record system, regardless of the original prescription date. We did not distinguish between newly initiated prescriptions versus ongoing/repeat prescriptions. Therefore, our findings reflect prescribing patterns by healthcare providers rather than actual medication dispensing or patient adherence to prescribed regimens. The collected data included: demographics, medical history, laboratory data, and current medications. To assess adherence to ADA guideline-recommended medications for people with diabetes and established ASCVD, the researchers developed a simple scoring system. Individuals received 1 point each if they were prescribed: a high-intensity statin (atorvastatin 40–80 mg or rosuvastatin 20–40 mg); an ACEi or ARB; a SGLT2i or GLP-1RA. Hence, the composite guidelines adherence score could range from 0 points (indicating no prescription of any recommended medication class) to 3 points (indicating prescription of all three recommended medication classes: high-intensity statin + ACEi/ARB + SGLT2i/GLP-1RA). This scoring scale serves as a simple metric to quantify adherence to ADA standards and facilitates the monitoring of prescribing practices. Each participant’s medication regimen was assessed independently by two researchers, with any discrepancies resolved through discussion and revisiting of the individual’s medication records.

For the purposes of this study, ‘prescribed’ medications were defined as those documented as active prescriptions in the electronic medical records system at the time of data extraction. This includes medications that were electronically prescribed by PHCC physicians and recorded in the patient's current medication list within the electronic health record system. We did not distinguish between newly initiated prescriptions versus ongoing/repeat prescriptions, nor did we assess whether prescriptions were actually filled at pharmacies or taken by patients.

### Data analysis

Data analysis was conducted using SPSS version 27. Descriptive summaries were generated using mean and standard deviation (SD) for continuous variables, and frequencies and percentages for categorical variables. The Chi-square test was performed to assess association within each categorical variable across the different EBM recommended classes (ACEi/ARBs, high-intensity statins, and SGLT2i/GLP-1RA), and across the prescribing categories (0–1 score and 2–3 score). For continuous variables, an independent *t*-test was used to evaluate mean differences between the two scoring categories. A binary logistic regression model was performed to assess the association of different variables on the likelihood that a person would receive EBM recommended medications (score 0–1 vs. score 2–3). Odds ratios with 95% confidence intervals were calculated for all logistic regression analyses. *P*-values < 0.05 were considered significant in the study’s analyses.

### Ethical considerations

The study protocol was reviewed and approved by the PHCC Research Committee in Qatar (reference number PHCC/DCR/2022/04/020). Since this was a retrospective analysis of routinely collected electronic medical records, the requirement for informed consent was waived by the Institutional Review Board. All data were fully de-identified prior to analysis, with access restricted to the Principal Investigator; no patient identifiers were accessible to the broader research team.

## Results

The records of 2,870 individuals with type 2 diabetes and ASCVD in Qatar’s PHCC centres were extracted. Only 10% of these people were prescribed high-intensity statins while 29.2% and 0.3% were prescribed moderate and low intensity statins, respectively, 12% were prescribed SGLT2i or GLP-1RA, and 56% were prescribed ACEI or ARB. Table 1 provides the characteristics of the study subjects. The average age of the participants was around 50 years, and 66% were male. The majority (91.3%) of the subjects were non-Qatari, with South Asians comprising the largest group (52.6%). The average BMI is 30.7 kg/m², indicating an overall prevalence of obesity, with 14.7% overweight, 11.8% class I obese, 5.8% class II obese, and 3% extremely obese.

**Table 1.** Characteristics of patients with diabetes and ASCVD attending primary care in Qatar stratified by evidence based medication use (*N* = 2,870).

Individual characteristic	Overall, Total=2870 *n* (%)	Receiving high-intensity statins, Total=292 *n* (%)	*P* value	Receiving ACEi or ARB, Total=1617 *n* (%)	*P* value	Receiving SGLT2i or GLP-RA, Total=357 *n* (%)	*P* value
**Age in years mean (SD)**	50.46 (9.77)	50.18 (10.39)		49.63 (9.68)		50.26 (10.03)	
**Sex**							
Male	1,894 (66.00)	217 (11.44)	0.001	1036 (54.61)	0.087	253 (13.34)	0.038
Female	976 (34.00)	75 (7.68)		581 (59.53)		104 (10.75)	
**Nationality**							
Qatari	250 (8.70)	31 (12.40)	0.223	127 (50.80)	0.032	47 (18.80)	0.001
Non-Qatari	2620 (91.30)	216 (8.24)		1517 (57.90)		310 (11.83)	
**Origin**							
South Asia	1510 (52.60)	143 (9.47)	0.015	875 (57.94)	0.020	182 (12.05)	0.035
Western Asia	546 (19.90)	70 (12.82)		293 (53.66)		88 (16.11)	
Southeast Asia	447 (15.60)	32 (7.16)		271 (60.63)		43 (9.62)	
North Africa	267 (9.30)	35 (13.11)		155 (58.05)		32 (11.99)	
Other	100 (3.50)	12 (12.00)		20 (20.00)		12 (12.00)	
**Smoking**							
Non-smoker	2512 (87.50)	243 (9.67)	0.115	1470 (58.52)	<0.001	303 (12.06)	0.043
Current smoker	239 (8.30)	31 (12.97)		111 (46.44)		31 (12.97)	
Former smoker	112 (3.90)	17 (15.18)		60 (53.57)		23 (20.54)	
Unknown	7 (0.20)	1 (14.28)		3 (42.86)		0	
**BMI** kg/m^2^ **mean (SD) (*n*=1186)**	30.66 (5.83)	31.15 (5.82)		31.36 (5.76)		29.74 (4.77)	
Underweight (<18.5)	2 (0.10)	0	0.438	1 (50.00)	0.021	0	0.620
Normal (18.5 – 24.9)	172 (6.00)	24 (13.95)		72 (41.86)		18 (10.47)	
Overweight (25 – 29.9)	421 (14.7)	52 (12.35)		225 (53.44)		56 (13.30)	
Obesity class I (30 – 34.9)	339 (11.8)	40 (11.80)		193 (56.93)		41 (12.09)	
Obesity class II (35 – 39.9)	167 (5.80)	16 (9.58)		85 (50.90)		18 (10.78)	
Extreme obesity (>40)	85 (3.00)	5 (5.88)		52 (61.18)		6 (7.06)	
**Comorbidities**							
Hypertension	2708 (94.40)	263 (9.71)	0.001	1626 (60.04)	<0.001	342 (12.63)	0.207
Dyslipidaemia	1072 (37.40)	207 (19.31)	<0.001	714 (66.60)	<0.001	160 (14.92)	0.002
Heart failure	52 (1.80)	9 (17.31)	0.086	16 (30.77)	<0.001	4 (7.69)	0.295
Chronic kidney disease	288 (10.00)	28 (9.72)	0.789	151 (52.43)	0.090	28 (9.72)	0.141
Myocardial Infarction	306 (10.70)	74 (24.18)	<0.001	107 (34.97))	<0.001	42 (13.73)	0.464
Peripheral vascular disease	8 (0.30)	0		3 (37.5)	0.299	2 (25.00)	0.281
**Diabetes complications**							
Retinopathy	57 (2.00)	6 (10.53)	0.929	29 (50.88)	0.104	11 (19.30)	0.113
Neuropathy	33 (1.10)	4 (12.12)	0.769*	20 (60.61)	0.728	6 (18.18)	0.315
Diabetic foot	4 (0.10)	0		2 (50.00)	0.571*	1 (25.00)	0.446
**Blood pressure mean (SD)**							
Systolic blood pressure mmHg	141.88 (20.39)	138.42 (19.50)		140.28 (18.27)		139.95 (18.32)	
Diastolic blood pressure mmHg	84.62 (11.67)	83.36 (10.55)		83.57 (10.43)		83.59 (10.77)	
**Laboratory readings mean (SD)**							
**HbA1c % (*n*=2011)**	**7.58 (1.81)**	**7.36 (1.76)**		**7.14 (1.57)**		**8.34 (1.78)**	
< 7 %	965 (48.00)	101 (10.50)	0.193	631 (65.40)	0.268	66 (6.80)	<0.001
7 – 7.99 %	422 (21.00)	44 (10.40)		298 (70.60)		61 (14.50)	
8 – 9 %	227 (11.30)	27 (11.90)		156 (68.70)		49 (21.60)	
> 9 %	397 (19.70)	57 (14.40)		265 (66.80)		119 (30.00)	
Fasting glucose mmol/L (*n*=1367)	8.55 (3.36)	8.33 (3.10)		8.10 (3.30)		9.62 (3.80)	
Total cholesterol mmol/L (*n*=1975)	5.04 (1.18)	5.36 (1.59)		5.05 (1.14)		5.08 (1.32)	
Triglycerides mmol/L (*n*=1974)	2.04 (1.34)	2.03 (0.98)		1.85 (0.93)		1.94 (0.97)	
HDL mmol/L (*n*=1937)	1.15 (0.28)	1.21 (0.31)		1.17 (0.28)		1.17 (0.28)	
LDL mmol/L (*n*=1888)	2.93 (1.02)	2.95 (0.91)		2.97 (0.98)		2.88 (1.06)	
**eGFR** mL/min/1.73m2 **mean (SD) (*n*=861)**	88.25 (23.36)	88.76 (23.37)		88.19 (22.16)		87.27 (24.50)	
<15	2 (0.10)	1 (50.00)	0.303	0	0.309	0	0.343
15 – 29	7 (0.20)	0		4 (57.14)		1 (14.28)	
30 – 60	65 (2.30)	8 (12.31)		38 (58.46)		13 (20.00)	
>60	787 (27.40)	96 (12.20)		488 (62.01)		98 (12.45)	
**Number of medications taken mean (SD)**	4.61 (4.10)	7.87 (4.44)		6.14 (4.04)		7.82 (4.96)	
≤ 3 medications	1347 (46.90)	41 (3.00)	<0.001	470 (34.90)	<0.001	63 (4.70)	<0.001
4 – 5 medications	529 (18.40)	60 (11.30)		383 (72.40)		71 (13.40)	
6 – 7 medications	399 (13.90)	57 (14.30)		300 (75.20)		69 (17.30)	
8 – 10 medications	345 (12.00)	61 (17.70)		281 (81.40)		74 (21.40)	
≥ 11 medications	250 (8.70)	73 (29.20)		219 (84.00)		80 (32.00)	
**PHCC region (*n*=2852)**							
Western region	882 (30.70)	83 (9.41)	0.661	526 (59.64)	0.091	113 (12.81)	0.068
Central region	1152 (40.10)	120 (10.42)		634 (55.03)		994 (86.28)	
Northern region	818 (28.50)	87 (10.64)		478 (58.44)		734 (89.73)	

*P* value was calculated using Chi-square.

*Reported using Fisher Exact test.

Only 37.4% of people were documented to have dyslipidaemia, while 94.4% had hypertension. Diabetes complications like retinopathy (2%) and neuropathy (1.1%) were relatively low. The mean HbA1c was 7.6%, indicating overall good glycaemic control. However, the percentage was slightly lower in individuals on high-intensity statins (7.4%) compared to those on SGLT2i or GLP-1 RA (8.3%).

The proportion of males receiving high-intensity statins and novel antidiabetic agents (SGLT2i or GLP-1RA) was significantly higher than that of females (11.4% vs. 7.7%; *p* < 0.001, and 13.3% vs. 10.8%; *p* = 0.038, respectively). Qatari individuals were more likely to receive novel antidiabetic agents compared to non-Qataris (18.8% vs. 11.8%; *p* = 0.001), whereas non-Qataris were more likely to receive ACEi/ARB as compared to Qataris (57.9% vs. 50.8%; *p* = 0.032). Patients with hypertension or dyslipidaemia had a higher likelihood of receiving ACEi/ARBs compared to those without these conditions (both *p* < 0.001). Furthermore, as shown in Table 2, individuals who were male and having hypertension, dyslipidaemia, retinopathy, or higher HbA1c were more likely to be prescribed EBM recommended medications (a score of 2–3). Table 3 provides details on the person characteristics based on the EBM prescribing score.

**Table 2.** Comparison of patient characteristics based on Evidence-Based Medication (EBM) Prescribing Scores in Patients with Diabetes and ASCVD in Qatar Primary Care.

Individual characteristic	Score 0–1, *n*=2430 (SD/%)	Score 2–3, *n*=440 (SD/%)	*P* value
**Age in years mean (SD)**	50.21 (9.79)	51.83 (9.57)	0.001
**Sex**			
Male	1578 (83.3)	316 (16.7)	0.005
Female	852 (87.3)	124 (12.7)	
**Nationality**			
Qatari	207 (82.8)	43 (17.2)	0.408
Non-Qatari	2223 (84.8)	397 (15.2)	
**Origin**			
South Asia	1282 (84.9)	228 (15.1)	0.152
Western Asia	448 (82.1)	98 (17.9)	
Southeast Asia	392 (87.7)	55 (12.3)	
North Africa	222 (83.1)	45 (16.9)	
Other	86 (86.0)	14 (14.0)	
**Smoking**			
Non-smoker	2132 (84.9)	380 (15.1)	0.206
Current smoker	203 (84.9)	36 (15.1)	
Former smoker	88 (78.6)	24 (21.4)	
Unknown	7 (100)	0	
**BMI** kg/m^2^ **mean (SD) (*n*=1186)**	30.81 (5.97)	29.70 (4.87)	0.020
Underweight (<18.5)	2 (100)	0	0.156
Normal (18.5 – 24.9)	148 (86.0)	24 (14.0)	
Overweight (25 – 29.9)	349 (82.9)	72 (17.1)	
Obesity class I (30 – 34.9)	288 (85.0)	51 (15.0)	
Obesity class II (35 – 39.9)	150 (89.8)	17 (10.2)	
Extreme obesity (>40)	78 (91.8)	7 (8.2)	
**Comorbidities**			
Hypertension	2283 (84.3)	425 (15.7)	0.027
Dyslipidaemia	821 (76.6)	251 (23.4)	<0.001
Heart failure	46 (88.5)	6 (11.5)	0.444
Chronic kidney disease	244 (84.7)	44 (15.3)	0.979
Myocardial Infarction	245 (80.1)	61 (19.9)	0.023
Peripheral vascular disease	6 (75.0)	2 (25.0)	0.353
**Diabetes complications**			
Retinopathy	41 (71.9)	16 (28.1)	0.014
Neuropathy	24 (72.7)	9 (27.3)	0.055
Diabetic foot	3 (75.0)	1 (25.0)	0.486
**Blood pressure mean (SD)**			
Systolic blood pressure mmHg	141.94 (20.39)	141.56 (20.4)	0.729
Diastolic blood pressure mmHg	84.73 (11.58)	83.98 (12.2)	0.234
Laboratory readings mean (SD)*			
HbA1c % (*n*=2011)	7.45 (1.77)	8.19 (1.84)	<0.001
Fasting glucose mmol/L (*n*=1367)	8.38 (3.30)	9.31 (3.53)	<0.001
Total cholesterol mmol/L (*n*=1975)	5.04 (1.11)	5.09 (1.43)	0.401
Triglycerides mmol/L (*n*=1974)	1.99 (1.22)	2.30 (1.77)	<0.001
HDL mmol/L (*n*=1937)	1.15 (0.27)	1.14 (0.28)	0.416
LDL mmol/L (*n*=1888)	2.95 (0.97)	2.82 (1.20)	0.074
**eGFR** mL/min/1.73m2 mean (SD) (*n*=861)*	88.25 (23.24)	88.23 (24.05)	0.990
<15	2 (100)	0	0.256
15 – 29	7 (100)	0	
30 – 60	50 (76.9)	15 (23.1)	
>60	662 (84.1)	125 (15.9)	
**PHCC region (*n*=2852)**			
Western region	741 (84.0)	141 (16.0)	0.616
Central region	974 (84.5)	178 (15.5)	
Northern region	701 (85.7)	117 (14.3)	

*P* values calculated using chi-square.

*P value calculated using independent *t*-test.

**Table 3.** Logistic regression analysis for evidence-based medication prescribing (Score 2–3 vs 0–1).

Variable	Odds	95% Confidence Interval	*P*-value
Male gender (ref: female)	1.64	1.37 – 1.79	0.001
Dyslipidaemia (ref: no)	1.56	1.29 – 1.73	<0.001
HbA1c (per 1% increase)	1.38	1.11 – 1.72	<0.001
**Variables Removed During Stepwise Selection:**			
Age (continuous)			0.420
Qatari nationality (ref: non-Qatari)			0.353
BMI (continuous)			0.226
Hypertension (ref: no)			0.264
Retinopathy (ref: no)			0.298
Neuropathy (ref: no)			0.099
Chronic kidney disease (ref: no)			0.168
Myocardial infarction (ref: no)			0.321

Model significance (*χ*^2^ (19) = 60.23, *p*<0.001), AUC = 0.678 (fair discriminative ability), Number of patients with complete data = 860 (30.0% of total cohort).

The logistic regression analysis included patients with complete data for all variables. The model demonstrated statistically significance (*χ*^2^ (19) = 60.23, *p* < 0.001), indicating its reliability in predicting EBM scoring. The model explained 16.3% of the variance in EBM scoring (Nagelkerke R^2^). Being a male (odds ratio [OR] 1.64, 95% confidence interval [CI] 1.37–1.79), a higher HbA1c (OR 1.38 per 1% increase, 95% CI 1.11–1.72), and a diagnosis of dyslipidaemia (OR 1.56, 95% CI 1.29–1.73) were associated with significantly higher odds of receiving EBM recommended medications. Other variables examined did not significantly contribute to the odds of receiving EBM recommended medications.

## Discussion

This study provides valuable insights into the prescribing trends of cardioprotective medications among patients with type 2 diabetes and established ASCVD who received care at primary care level in Qatar. The findings suggest that there is potential to improve the prescribing of these essential medications, given that only three out of 20 eligible individuals received two or three classes of the cardioprotective agents. Despite guidelines and evidence supporting the use of novel antidiabetic medications such as SGLT2i and GLP-1RA, only 12% of eligible patients in Qatar received these treatments. In contrast, more than half of eligible patients received older agents such as ACEi or ARBs, surpassing the 45% rate reported in other studies. However, there is still room for improvement (Nelson et al., 2022). Similar trends have been observed in other countries as well. For instance, a study conducted at Saudi Arabia’s primary care setting found that only 14% of individuals with established ASCVD and diabetes were prescribed SGLT2i or GLP-1RA, highlighting the need for enhanced specialist care and adherence to guidelines to achieve better cardiovascular outcomes (Korayem et al., 2022). Similarly, a USA-based study spanning 2015 to 2019indicated a rise in the utilisation of GLP-1RA (from 6.3% to 13.3%) and SGLT2i (from 6.2% to 14.4%). Despite this increase, a significant portion of patients did not receive these medications, with those aged over 65 years being particularly disadvantaged (Mody et al., 2022). It seems that the prescribing rates observed in Qatar are comparable to those in other countries, where a disparity exists between the guidelines and real-world clinical practice. Adherence to guidelines recommendations through appropriate prescribing will ultimately result in improved clinical outcomes (ElSayed et al., 2023).

An important consideration in interpreting our findings is the temporal relationship between guideline publication and clinical implementation. While landmark cardiovascular outcome trials for SGLT2i and GLP-1RA were published between 2015–2018, formal ADA guideline recommendations were predominantly issued in 2020–2022. Our 2021 data collection represents a transitional period where updated guidelines had recently been published but may not have been fully disseminated, adopted, or implemented in routine clinical practice. This implementation lag is well-recognized in evidence-based medicine and likely contributes to the suboptimal prescribing rates observed in our study (Beauchemin et al., 2019; Thoonsen et al., 2025). Therefore, our findings should be interpreted as baseline data capturing prescribing patterns during the early implementation phase of updated guidelines, rather than representing established practice patterns.

This study identified certain patient characteristics influencing the likelihood of receiving evidence-based cardioprotective medications. Specifically, being male, having higher HbA1c, and being diagnosed with dyslipidemia were associated with increased odds of being prescribed recommended agents. However, these factors collectively explained only 16% of the variability in prescribing patterns, indicating that the of the influencing factors were not accounted for. This underscores the complex and multifaceted nature of clinical inertia in clinical practice. Identifying factors associated with lower medication utilisation may highlight specific subgroups that may benefit from targeted quality improvement initiatives. For example, interventions targeting healthcare providers could mitigate inadvertent biases in prescribing practices for younger versus older adults. Efforts should also ensure that women with cardiovascular risk factors receive recommended medications as their male counterparts. Further explanatory studies, perhaps using qualitative approach, could further elucidate and clarify the reasons behind the under-prescribing of the cardioprotective agents in primary care settings.

The variations in the prescribing rates of novel antidiabetic agents (SGLT2i and GLP-1RA) relative to cheaper ACEi/ARBs between Qatari and non-Qatari people can be attributed to variations in insurance coverage in Qatar. Under the Ministry of Public Health (MoPH) coverage system, all medications are fully covered for Qataris in public sector hospitals and primary care clinics. However, at the time of this study, non-Qataris received up to 80% coverage, with a 20% co-payment required (including for high-cost medications), which is typically paid out of pocket (Nadeem et al., 2021). It is important to note the recent changes in health insurance scheme implemented in Qatar. The MoPH has recently mandated that all non-Qataris living in Qatar must now receive medical coverage from their employers, while visitors to Qatar are required to have private insurance. This evolving insurance landscape in Qatar suggests a potential increase in the future utilisation of novel antidiabetic agents among citizen and residents of Qatar (New mandatory health insurance system introduced in Qatar, 2023).

While our findings indicate low prescribing rates for guideline-recommended medications, it is important to acknowledge that some patients may have had valid clinical contraindications that would make these therapies inappropriate. Based on available data from our study population, we can estimate potential contraindications: for SGLT2i, approximately 74 patients (2.6%) had severe chronic kidney disease (eGFR <30 mL/min/1.73m²) representing a contraindication; for ACE inhibitors/ARBs, patients with hyperkalemia or bilateral renal artery stenosis would be contraindicated, though we lack systematic data on these conditions; for high-intensity statins, active liver disease would represent a contraindication, but this information was not systematically captured in our dataset. Even accounting for these potential contraindications, the vast majority of our study population would remain eligible for these therapies, suggesting that clinical contraindications alone cannot explain the observed low prescribing rates.

Clinical inertia, defined as the reluctance of a healthcare provider to optimise treatment or initiate evidence-based medicine is a significant issue in healthcare (Lewinski et al., 2022). There are several factors that contribute to the clinical inertia observed in the prescribing of cardioprotective drugs. These include old age, perhaps due to the potential frailty; the presence of multiple comorbidities; concerns about hypoglycaemia and other adverse effects; patient preferences to avoid multiple medications; prescriber-related factors such as specialty and limited awareness of the cardiovascular benefits of the medications, and the physician reluctance to switch established medications. Other non-medical factors that may contribute to under-prescribing include high medication costs, formulary restrictions, and insurance coverage (Korayem et al., 2022; Mody et al., 2022). In Qatar, Al-Dahshan et al. described the reasons for inappropriate prescribing, which can be extrapolated to under-prescribing. The investigators found that patients with polypharmacy were over six times more likely to have inappropriate medications prescribed compared to those without polypharmacy. Furthermore, having specific comorbid conditions, including diabetes, hypertension, cardiovascular diseases, asthma, gastroesophageal reflux disease (GERD), and arthritis, were significant predictors of inappropriate prescriptions. The risk of inappropriate prescribing increased with the presence of multiple comorbidities (Al-Dahshan & Kehyayan, 2021).

The published literature suggested solutions to combat clinical inertia in healthcare settings. One effective approach is expanding the role of pharmacists in the primary care setting. Including pharmacists on care teams of patients with type 2 diabetes has been shown to promote EBM prescribing, improve glycemic control, and reduce the risks of adverse cardiovascular outcomes (Lewinski et al., 2022; Trueman et al., 2023). In Qatar, pharmacists currently play a role in primary care, often focusing on medication dispensing, patient education, and medication safety. However, there is potential for greater integration of pharmacists into primary care settings, where they can contribute more extensively to patient care through medication management, chronic disease management, and collaborative practice with other healthcare professionals. The COVID-19 pandemic highlighted the important contributions pharmacists can make within primary care settings in Qatar. During the pandemic, pharmacists demonstrated their capability in expanded roles, including education, medication management, vaccination efforts, and other clinical responsibilities (Primary Health Care Corporation, 2022). There are now ongoing plans in the country to further integrate clinical pharmacists into PHCC and expand their roles within primary care (General communication office, 2008). Some specific initiatives include conducting medication reviews for chronic conditions like diabetes, providing one-on-one medication counselling, monitoring for drug interactions and side effects, assisting with dose adjustments, and providing evidence-based recommendations to prescribing physicians (General communication office, 2008; Jebara et al., 2021). Indeed, pharmacists are uniquely positioned to help address challenges like under-prescribing of cardioprotective agents, which can contribute to reducing cardiovascular risk and its associated complications (Ballantyne, 2007; Riordan et al., 2016; Trueman et al., 2023).

Another effective approach to combat clinical inertia is academic detailing, where expert educators provide clinicians with individualised education on guidelines and best practices (Wells et al., 2016). Additionally, implementing performance metrics and prescription quality measures can incentivize appropriate prescribing practices and hold providers accountable (Chawla et al., 2001). Clinical decision support tools integrated into electronic health records can also provide point-of-care reminders and alerts to clinicians about indicated medications (Armando et al., 2023). Overall, multifaceted approaches combining provider, team, and system-level strategies hold the greatest promise for overcoming clinical inertia and optimising the utilisation of cardioprotective medication use (Soumerai et al., 2005). Further research is warranted on the comparative effectiveness of different interventions to address this important gap in care and improve healthcare outcomes.

Our single-year cross-sectional design represents an important limitation, as longitudinal studies examining prescribing trends over multiple years provide more robust evidence of guideline implementation and uptake patterns. For example, Raat et al. demonstrated the value of multi-year analysis (2019–2023) in Belgian primary care, showing evolving trends in type 2 diabetes medication use and guideline adherence over time (Raat et al., 2025). Such longitudinal approaches can distinguish between persistent clinical inertia and gradual improvement following guideline updates. Additionally, the study is limited by the inherent potential biases in retrospective studies that rely on obtaining data from medical records, such as discontinuity and missing or incomplete data. Our dataset lacked systematic documentation of contraindications, drug allergies, or intolerances that would represent clinically appropriate reasons for not prescribing guideline-recommended medications. This limitation prevents us from distinguishing between inappropriate under-prescribing due to clinical inertia versus appropriate clinical decision-making based on patient-specific contraindications. We also lacked data on the total PHCC population in 2021, so prevalence estimates could not be calculated; our analysis was restricted to patients with type 2 diabetes and ASCVD identified in electronic health records. Our analysis was also limited by the assessment of prescribed rather than dispensed medications, lack of systematic documentation of contraindications or drug allergies, as well as lack of a classification system that reduces reporting errors. In fact, our study is that only 30% of patients had complete data for inclusion in the logistic regression analysis. Also, we did not conduct separate logistic regression analyses for ‘older’ (statins, ACE inhibitors/ARBs) versus ‘newer' (SGLT2 inhibitors, GLP-1 receptor agonists) medications. The relatively low prescribing rate of SGLT2 inhibitors and GLP-1 receptor agonists (12%) would render subgroup-specific models underpowered and potentially unstable. Additionally, the logistic regression analysis was restricted to patients with complete data for all variables, which further diminished the sample size available for any stratified analyses. Therefore, we combined all classes into a composite outcome to enhance statistical power and provide a broader perspective on evidence-based prescribing. Future studies utilising larger or multi-year datasets would enable stratified analyses to differentiate between older and newer cardioprotective medications, yielding deeper insights into evolving adoption patterns. Therefore, it is important to interpret the results with caution. Despite these limitations, the findings of this study are consistent with other real-world evidence that also highlights the treatment gaps. This consistency across different studies underscores the significance of the findings and the persistent challenges in ensuring optimal medication use among patients with diabetes and ASCVD. Future research should aim to address these limitations through more robust study designs and improved data collection methods.

## Conclusion

In conclusion, this study provides for the first time the evidence of under-prescribing of recommended cardioprotective medications among individuals with diabetes and ASCVD in Qatar. Only a small proportion of eligible patients received evidenced-based combination therapies including high-intensity statins, ACEi/ARBs, and novel antidiabetic agents like SGLT2i or GLP-1RA. Aligning real-world prescribing practices with established guidelines has great potential to improve cardiovascular outcomes for a large number of individuals in Qatar. However, given our observational design and identified limitations, longitudinal that examine prescribing patterns over time and implementation studies that systematically account for contraindications, and access constraints are needed to inform evidence-based policy interventions. Such studies would better distinguish between appropriate clinical decision-making and modifiable barriers to guideline implementation. Nonetheless, this study underscores the urgent need for healthcare providers and policymakers to implement comprehensive and holistic strategies to overcome clinical inertia and ensure that patients receive appropriate medications for cardiovascular protection. These efforts are crucial for advancing healthcare practices in Qatar and ensuring better management of cardiovascular health among its population.
